# Validity of Measured vs. Self-Reported Weight and Height and Practical Considerations for Enhancing Reliability in Clinical and Epidemiological Studies: A Systematic Review

**DOI:** 10.3390/nu16111704

**Published:** 2024-05-30

**Authors:** Khadijah Fayyaz, Mo’ath F. Bataineh, Habiba I. Ali, Ali M. Al-Nawaiseh, Rami H. Al-Rifai’, Hafiz M. Shahbaz

**Affiliations:** 1 Department of Nutrition and Health, College of Medicine and Health Sciences, United Arab Emirates University, Al Ain P.O. Box 15551, United Arab Emirates; 2021-mphil-1162@uvas.edu.pk (K.F.); habali@uaeu.ac.ae (H.I.A.); shahbaz@uaeu.ac.ae (H.M.S.); 2 Department of Food Science and Human Nutrition, University of Veterinary and Animal Sciences, Lahore 54000, Pakistan; 3 Department of Sport Rehabilitation, Faculty of Physical Education and Sport Sciences, The Hashemite University, Zarqa 13133, Jordan; nawaiseh@hu.edu.jo; 4 Department of Public Health Institute, College of Medicine and Health Sciences, United Arab Emirates University, Al Ain P.O. Box 15551, United Arab Emirates; rrifai@uaeu.ac.ae

**Keywords:** validity, weight, height, self-reported, reliability, systematic review

## Abstract

Self-reported measures of height and weight are often used in large epidemiological studies. However, concerns remain regarding the validity and reliability of these self-reported measures. The aim of this systematic review was to summarise and evaluate the comparative validity of measured and self-reported weight and height data and to recommend strategies to improve the reliability of self-reported-data collection across studies. This systematic review adopted the PRISMA guidelines. Four online sources, including PubMed, Medline, Google Scholar, and CINAHL, were utilised. A total of 17,800 articles were screened, and 10 studies were eligible to be included in the SLR based on the defined inclusion and exclusion criteria. The findings from the studies revealed good agreement between measured and self-reported weight and height based on intra-class correlation coefficient and Bland–Altman plots. Overall, measured weight and height had higher validity and reliability (ICC > 0.9; LOA < 1 SD). However, due to biases such as social pressure and self-esteem issues, women underreported their weight, while men overreported their height. In essence, self-reported measures remain valuable indicators to supplement the restricted direct anthropometric data, particularly in large-scale surveys. However, it is essential to address potential sources of bias.

## 1. Introduction

Anthropometric measurements, specifically height and body weight, are important indicators of health in clinical and community contexts [1]. The assessment of an individual’s general health and nutritional status frequently involves the use of these anthropometric measures. Moreover, to effectively execute public health initiatives, it is important to monitor changes in the burden of obesity and underweight in general populations by means of body composition based on weight and height parameters [2].

In general, directly measured height and weight represent the preferred method for calculating BMI. However, to collect these metrics, measurements must be carried out by qualified personnel utilising validated measuring tools, which can be expensive and labour-intensive [3]. In large-scale surveillance-based research, these limitations are compounded significantly. Therefore, to determine BMI in such community-oriented studies, clinicians and epidemiologists utilise participants’ self-reported weight and height [4]. This is because employing questionnaires or interviews to gather data is both easy and efficient. Several epidemiological studies have used these self-reported methods in place of precise measurement methods to obtain information on height and weight [2,5,6,7].

Often, data on the prevalence of overweight and obesity in the population of many countries are based on self-reported anthropometrics, as they represent a less labour-intensive, less time-consuming, and cheaper substitute to objective measures of these indices, when sampling large groups [4]. However, there are several reports of poor validity and reliability of these self-report measures across studies. A large international study analysing misreporting patterns of height and weight with 14,650 participants aged 50 and over in China, India, Russia, and South Africa has revealed that self-reported weight was higher than measured weight in China (up to 7.6 kg in those aged 80 and over) but lower in India, Russia, and South Africa (usually around 1 kg or less). Furthermore, the same study showed that the overall self-reported height was lower than the measured height in India and South Africa and higher in China and Russia [8]. Additionally, self-reported weight and height versus direct measurements yielded good correlations across a variety of validity research studies [3].

Furthermore, measurement biases undermine the systematic reporting of anthropometrics; therefore, self-reported weight and height tend to differ from measured weight and height. When looking into the reliability of self-reported anthropometrics, research has shown that people tend to exaggerate their height and underestimate their weight regardless of sex [2]. Self-reported height and weight are understandably found to underestimate the prevalence of obesity, whereas differences in underweight prevalence are unclear. Since weight and height are self-reported, the BMI derived from self-reported data is often lower than the BMI derived from measured data, since weight is typically underestimated and height is overestimated [9]. Consequently, the relative risks of illnesses linked to differential BMI can be skewed by variations in self-reported and measured weight and height [10].

A previous study found that height, weight, and BMI were overreported with different mean reporting errors. The direction of false reporting for height was the same across all body sizes; however, only the underweight showed the overreporting and overestimation of weight and BMI [1]. Another study revealed that compared with the measured height, which was 0.71 cm more for women and 0.59 cm greater for men, the self-reported weight was 0.67 kg less for women and 0.55 kg less for males. Consequently, when compared with the measured BMI, the self-reported BMI was understated (0.35 kg/m^2^ lower for males and 0.49 kg/m^2^ lower for women) [11].

Inaccuracies in self-reported height and weight lead to incorrect interpretations of BMI, which can impact health program planning and monitoring [12]. Moreover, resource scarcity can also necessitate the use of self-reported-data collection methods. Therefore, it is crucial to ensure the accuracy of the self-reported data, even if real-time measurements cannot be obtained [13]. The purpose of this systematic review is to summarise and evaluate the comparative validity of measured and self-reported weight and height data from different clinical and epidemiological studies and to recommend workable strategies to improve the reliability of anthropometric data collection considering the inconsistencies observed in terms of the validity of measured vs. self-reported data across studies.

## 2. Systematic Search

### 2.1. Study Design

This research implemented an evaluative approach with the use of systematic literature review (SLR). The PRISMA approach was followed to report this systematic review [14]. It is an evidence-based minimum set of reporting standards for systematic reviews and meta-analyses. A 4-phase flow diagram and a 27-item checklist make up the PRISMA statement [14]. The strategy used to conduct this SLR was appropriate, as it made it possible to extract and screen studies for the review. The PRISMA checklist is provided in Supplementary Table S1.

### 2.2. Eligibility Criteria for Study Inclusion

Included were studies, whether observational or experimental, that directly contrasted self-report and objective measures for the targeted aims. All study designs were eligible for inclusion in the systematic review, spanning cross-sectional, case–control, prospective, retrospective, and randomised controlled trials. Studies that used a stadiometer, tape measure, portable or balance beam scale, or any other method to measure weight or height were acceptable for inclusion. Similarly, self-reporting methods in any format (e.g., interviewer-administered and self-complete) were included. Reports submitted by proxy, in which one person answers on behalf of another, were not accepted for inclusion (Table 1).

### 2.3. Database Search Strategy and Identification of Eligible Studies

Online sources including PubMed, Medline, Google Scholar, and CINAHL were utilised to retrieve and compile articles for this systematic review. The Boolean operands, keywords, and their combinations were integrated into the advanced search features of the search engines in order to find pertinent contemporary literature studies and data in the form of published research connected to the topic. To acquire relevant search results, the Boolean operands (AND/OR) were explicitly indicated along with other research-related terms. Phrase searching was employed to locate and retrieve the major research studies by using keywords, which comprised (comparative validity OR validity comparison) AND (measured OR objective) AND (self-reported OR self-report) AND weight AND height AND (clinical OR medical) AND epidemiological AND (practical strategies OR interventions) AND reliability AND anthropometric data collection (Supplementary File).

After the initial database search, a meticulous screening process was conducted by two independent reviewers to retrieve relevant studies based on the defined inclusion criteria. Studies were screened based on the titles and abstracts to retrieve articles that aligned with the defined inclusion criteria. Those studies whose titles and abstracts did not match the inclusion criteria were excluded. Following the initial screening of studies, an independent assessment of the full texts of the studies was carried out by the reviewers to retrieve eligible studies.

### 2.4. Data Extraction Process

Studies were gathered by using the previously mentioned inclusion and exclusion criteria. The authors, publication years, study types, regions, and countries were among the data extracted from each eligible study, along with participant characteristics such as age, educational attainment, sample size, and methods for directly measuring weight and height (stadiometer and other types of measuring devices, such as tape measures or rulers). Other methods for gathering self-reported weight and height data included questionnaires completed by participants, surveys, on-site interviews, time intervals between collecting self-reported and directly measured data, sequence of measures, ethical considerations, and results.

Data extraction was performed by two independent reviewers to ensure accuracy and reliability in retrieving the relevant literature for the systematic review. Any points of disagreement between the reviewers were sorted through discussion and consensus. A third reviewer was consulted to resolve disagreements and obtain a conclusion in situations where consensus could not be reached. This way, transparent and reliable data extraction was ensured to include eligible studies in this systematic review.

### 2.5. Method of Quality Assessment of Studies

One of the essential steps in any type of systematic review is to conduct a critical or quality appraisal. In this systematic review, the Critical Appraisal Skilled Program tool (CASP, Link: https://casp-uk.net/casp-tools-checklists/, accessed on 15 April 2024) was used to qualitatively evaluate the risk of bias and assess the quality of the included studies. It is a general instrument for evaluating any qualitative research method. Ten questions make up the instrument, and each one focuses on a distinct methodological facet of a qualitative investigation. The tool’s questions enable the researcher to evaluate if the study procedures are suitable and whether the results are presented and significant. The CASP tool served as a criteria-based instrument for examining quality and methodological rigor of the qualitative studies. For the quality appraisal of quantitative research, JBI’s standardised critical appraisal tool was used.

### 2.6. Risk of Bias Assessment (RoB 2.0 and ROBINS-E)

Depending on study design, the risk of bias (RoB) in the included studies was examined and evaluated by using proven methods. For randomised controlled studies, the Cochrane Risk of Bias tool for Randomised studies (RoB 2.0) was employed. This tool focuses on several elements of research design by using a predetermined set of bias domains. Alternatively, for the non-randomised studies, RoB in Non-randomised Studies (ROBINS-E) was employed (Supplementary File).

## 3. Results

### 3.1. Database Search

**Figure 1:**
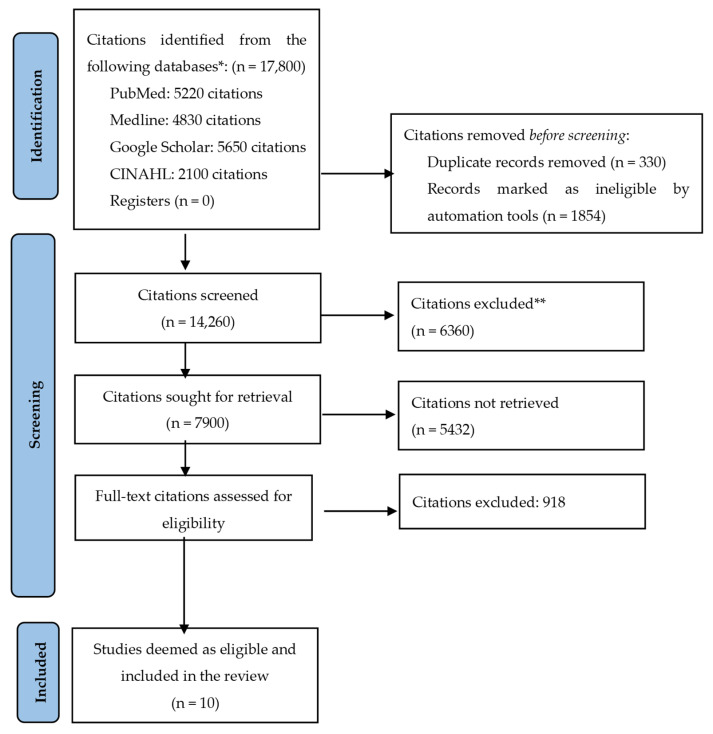
The PRISMA flow diagram depicting the identification, screening, and selection of studies for this systematic review. * Total studies retrieved from databases based on keyword search. ** Excluded studies that did not meet the inclusion criteria.

The database search yielded a total of 17,800 citations. After deduplication and a thorough evaluation against the set of eligibility criteria, a total of 10 studies were deemed eligible and included in this review. Figure 1 shows the PRISMA flow diagram depicting the identification, screening, and selection of studies for this systematic review.

### 3.2. The Scope of the Review and Characteristics of the Included Studies

Table 2 summarises the characteristics of the studies included in this systematic review. The 10 included studies were from Australia, Brazil (two studies), Croatia, Korea, Malaysia, Singapore, Turkey, and the United States (two studies). The included studies involved a range of research designs, including qualitative design, cross-sectional surveys, and randomised controlled trials. The demographics of the participants were similarly diverse, ranging from those who were 65 years of age and above to secondary-school children. Most studies had mixed sex representation in their sample sizes, which varied from 286 to 214,640 participants. The self-reporting techniques used included, self-administered, interview, and questionnaire surveys.

### 3.3. Findings

In their 2023 study, Chia et al. examined the consistency and accuracy of adult Malaysians’ self-reported weight and height in relation to their actual measured metrics. There were 0.4 kg and 0.4 cm discrepancies in the mean reported weight and height compared with the measured values [2]. Another study conducted in Brazil demonstrated good agreement (ICC > 0.9) between actual and reported values of height, weight, and computed BMI. Except for female height measurements, where the ICC was 0.8920, all the ICC values when weight, height, and BMI were classified by sex exceeded 0.9. The ICC values were more than 0.75, which is considered a high degree of agreement, when weight and height measurements were grouped by BMI. Males who were overweight had the lowest degree of agreement (0.7604) [15].

A study carried out in the United States showed that variations in BMI categories were caused by underweight and overestimation of height. Overestimating self-reported height and underestimating self-reported weight led to an underestimation of BMI [9]. In a similar vein, the study by Roystonn et al. (2021) showed that women estimated their height to be 0.35 cm greater, compared with 0.02 cm for men. Women reported being 0.95 kg underweight, compared with men who reported being underweight by 0.63 kg. The LOA values for both weight and height were less than 1 SD of the corresponding measured values, which were estimated to be 13.4 kg and 9.1 cm, respectively [17]. This indicates that there was usually good agreement between self-reported estimations and actual measurements.

A similar trend was observed in terms of age progression and self-reporting in the research by Ko et al. (2022). When compared with the measured values, the reported height values for men and women were 0.59 and 0.71 cm greater, respectively. In other words, males as well as females reported greater height values than their real measures as they grew older [11]. In a similar vein, the study by Fry et al. (2022) showed a similar trend, indicating that as males and females age, they tend to overestimate their weight and height [10].

In addition, in the research conducted by Štefan et al. (2019), Kappa data revealed that the underweight, normal, and overweight categories agreed well in girls and well in boys. For both boys and girls, the overweight status had the lowest sensitivity scores. For boys, the range of specificity values was 88.9% to 100%, while for girls, it was 50.0% to 93.0% [16]. Likewise, in the research by Beleigoli et al. (2019), the relationship between BMI and weight did not reach statistical significance [5]. Nevertheless, the difference between measured and reported height (*p* < 0.001) was 0.004 metres (SD 0.012). Less than 5% of the reported and measured values for height, weight, and BMI were outside of the confidence interval boundaries, according to the Bland–Altman plots [16].

Fry et al. (2022) argued that women underestimated weight by 0.75 kg more than men did. Higher educated individuals properly estimated their weight by 1.0 kg compared with the least educated individuals. Compared with people born in non-English-speaking countries, those born in Australia and other countries with a large English-speaking population underestimated weight by 1.4 kg. Persons whose language was not English overestimated their weight by 1.6 kg or overestimated it by 2.1 kg than persons whose first language was English or those whose second language was another language although they spoke English well, respectively. Those with diabetes underestimated their weight by 1.0 kg, and those with musculoskeletal disorders underestimated their weight by 0.8 kg [10].

In a population of 5445 men and 1905 women, for example, Hodge et al. (2020) reported that 12.7% of men and 14.4% of women misclassified their weight and height. It was shown that in a group of 1870 men and 2938 women, there were misinterpretation rates of 15.2% for women and 22.4% for men using higher thresholds for low BMI. When utilising self-reported BMI, 15% of men and 10% of women were classified in a different WHO category; the study’s degree of inaccuracy compares positively to these previous findings [9].

#### Subgroup Analysis: Gender-Based Variation

**Figure 2:**
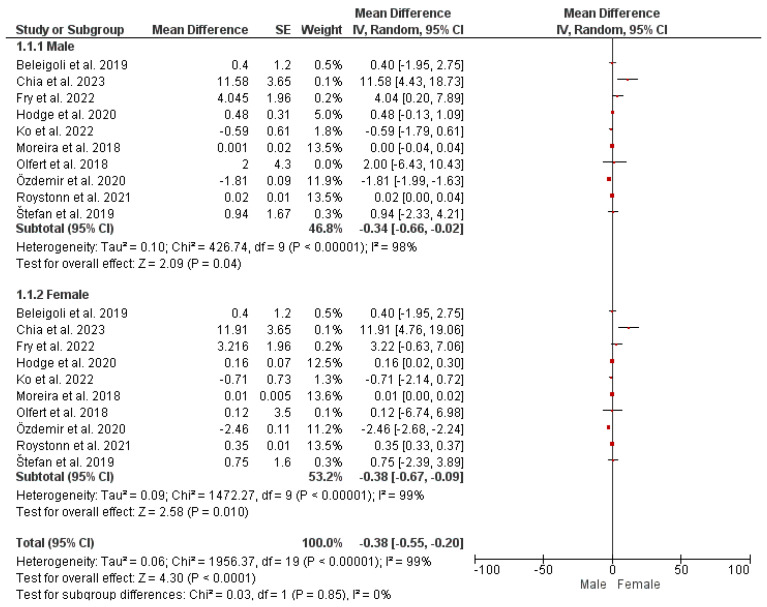
Gender-based differences in self-reported vs. measured height [2,5,6,9,10,11,15,16,17,18].

**Figure 3:**
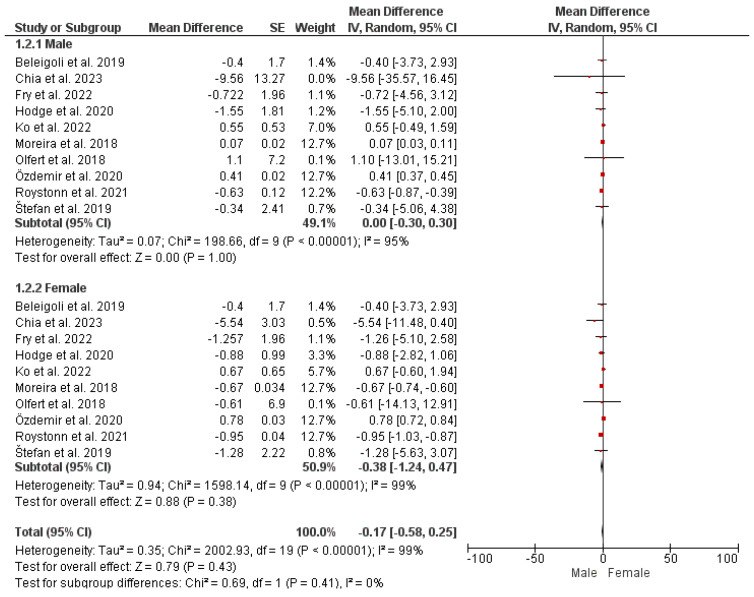
Gender-based differences in self-reported vs. measured weight [2,5,6,9,10,11,15,16,17,18].

To account for gender-based differences across studies in terms of self-reported and measured height and weight, a subgroup analysis was conducted and presented as a forest plot (Figure 2 and Figure 3). The findings from the analysis revealed that although there are gender-based variations in self-reported vs. measured height and weight, with women underreporting their weight and men overreporting their heights, there is no statistically significant difference (*p*-value > 0.05) in mean height discrepancies between male and female subgroups across the studies. Moreover, no heterogeneity was observed across studies, and the variation in mean height discrepancies between male and female subgroups was minimal according to the I-squared statistic (I^2^ = 0%; χ^2^ = 0.03).

Overall, these findings imply that gender-based differences may occur in self-reporting weight and height. However, these differences are non-significant with low heterogeneity across the studies.

## 4. Discussion

### 4.1. Discrepancies and Validity Concerns in Measured vs. Self-Reported Weight and Height

The majority of research studying the prevalence of obesity involves accurate measurements of height and weight [8,12,19]. Self-reported weight and height have been used in epidemiological studies because it has been shown that they are reliable, useful, and inexpensive alternatives to measurement methods. However, self-reported height and weight may not always be correct due to social influences and regional differences in health awareness [19,20].

Analogous research conducted in the USA, Japan, and England likewise showed a strong correlation between self-reported and actual BMI, weight, and height measures [1,9,21]. Despite the minor discrepancies in self-reported data seen at the individual level, this study showed a significant connection between measured and reported weight and height. Strong correlations (>0.9) were found between measured weight, height, and BMI and self-reported values, respectively. These results were consistent with those of other studies conducted in Asia, particularly in Japan [7,22,23,24].

Similarly, the weight values that the men and women self-reported were 0.55 kg and 0.67 kg lower than the weight that was really measured. The difference between self-reported and measured weight values was greater among middle-aged and older adults. Individuals who were older than 70 showed a significant reduction in the gap. In every age group, most women underreported weight compared with men [11].

### 4.2. Factors Influencing Validity and Reliability of Self-Reported Data

Factors such as age, sex, race, and socioeconomic level influence the degree to which height and weight are misreported [25]. According to a prior research study, adults often underestimate their body weight [20]. The difference between measured and self-reported weights has been found to vary depending on the respondent’s sociodemographic traits, such as age, sex, weight, and race/ethnicity [17,20,26]. For instance, compared with women of average weight, obese women often underreport their weight. Conversely, underweight women are more likely to underestimate their weight when compared with their peers of normal weight [20]. People may not voluntarily classify themselves as overweight or obese due to the poor social acceptability of being in such categories [27].

Empirical studies have shown that self-reported weight and height may vary in accuracy due to factors such as health awareness, social influences, and demographic characteristics [10,17]. The research populations’ demographic makeup most likely contributed to the various bias tendencies [2]. The common characteristics that contributed to the varying and dramatic findings in research were age, sex, and higher BMI categories.

Studies indicate that there may be considerable differences in the accuracy of self-reported anthropometric parameters based on race or ethnicity and sex [5,18]. It has been observed that women tend to underreport weight, while males prefer to overreport height. Moreover, research has also demonstrated that regardless of other sociodemographic factors, ethnic groups differ considerably in their underestimation of the incidence of overweight or obesity based on self-reported height and weight [17].

Men are informed on their most recent height and have their bodies measured when they turn 19 as part of the prerequisites for joining the army. But most women only take height measurements when they are in school, which causes discrepancies and inaccuracies between the measured and self-reported height levels. The research study also discovered that for males, the weight difference was favourable and grew with age. The discrepancy between self-reported and measured height was positive and grew with age for both sexes in the case of females [10]. When individuals with the lowest level of education were given the same information, the analysis revealed that males were more likely to overestimate their weight and women to underestimate it. In essence, the lower the level of education, the more the participants overestimated their height [10].

Comparable outcomes were associated with self-reported health in males and poverty in both sexes [15]. In the group of low poverty and the lowest level of education, both sexes overestimated their height more. Additionally, women who lived alone tended to exaggerate their height. There was a relationship between worse self-rated health and males as well as females overreporting their height. Age and, in the case of men, self-reported health were associated with higher levels of BMI underreporting. The correlation between significant overestimation of height for both sexes and weight for females and impaired cognition has been associated with psychological distress [9,15].

Moreover, significant differences in height (overestimation) across all the listed medical diseases were associated with musculoskeletal issues. Diabetes and musculoskeletal disorders were associated with an underestimation of body weight, consistent with a study showing that Catalans underestimated weight much less if they had (unspecified) chronic diseases. However, research conducted in the USA found that males who had osteoporosis overstated their weight [9].

The underestimation of weight was not significantly different between individuals born in the UK or Ireland and those born in Australia; however, those born in Eastern and Western Europe underestimated their weight more than those born in Australia [10]. This might be consistent with Australian-born individuals using healthcare services more frequently, which leads to a greater clinical emphasis on weight. An alternative reason might be the social elements that influence people’s habits, which in turn influence societal norms about the ideal body weight. Self-reported and measured weight differences have been associated with manual socioeconomic class membership in Britain [21].

Older people overestimate their height because they recollect having their height measured earlier in life before growing shorter due to changes in bone and muscle, whereas younger women prefer to underestimate their weight due to social desirability bias [21]. Ethnic differences also lead to differences in measured and self-reported weight and height. The Indians and Malays reported more discrepancies in their self-reported height and weight, leading to a higher likelihood of misreporting their true overweight or obesity status compared with the Chinese [17]. Likewise, non-Hispanic black American women reported greater weights compared with non-Hispanic white women [20,26].

The data reported in the literature support the hypothesis that there is a substantial correlation between age and height. Given that it has been shown that height is exaggerated more in situations when people show signs of cognitive decline, this discovery might be the result of both increased postural issues and cognitive loss associated with ageing and psychological distress [28]. Moreover, a longer time interval between the height measurements may lead to a larger overestimation. In contrast, women tend to overestimate height less frequently than men do. Given that osteoporosis has been associated with a decline in women’s overestimation of height, this would be consistent with the condition making clinicians more aware of women’s height [10].

According to a prior study by Štefan et al. (2019), university students had trouble in accurately identifying their body shape. Women thought they were overweight even though there was a low rate of obesity among students [16]. Many studies believe that women are under intense pressure to reduce their weight because they want to be lean to be accepted by society. The media’s promotion of thinness may reflect societal desirability, which in turn may influence a person’s desire to be lean. Changes in body perception are significantly influenced by the idealisation of thinness in the media and society at large. Eating habits, mass media, personal expectations, and cultural conventions may all have an impact on one’s perceived and actual weight. Other contributing causes might be the impact of Western society, the worry of not being loved by the other sex, or the anxiety of not finding clothing that fit one’s physique [16].

### 4.3. Implications in Epidemiological and Clinical Settings

Obtaining real measurements by qualified personnel using equipment that has been verified is a labour-intensive and expensive task that presents significant hurdles for large-scale data collection in epidemiological investigations. Undertaking population-specific evaluations of the self-reported data’s reliability to spot potential biases and demographic variables influencing the self-reported information’s reliability is one practical strategy for improving reliability [2].

In clinical settings, validity concerns regarding inaccurate anthropometric data could lead to misclassification of individuals’ nutritional status, potentially impacting the accuracy of clinical assessments and interventions. Empirical studies have shown that self-reported weight and height can be reliable for BMI calculation, providing a feasible and reliable measurement, especially in online or mass screening surveys in Malaysia [2]. Adult Malaysians’ self-reported heights and weights are accurate and trustworthy enough to be utilised as indicators in data gathering and research pertaining to public health. By using self-reported weight and height, obesity in individuals or the general population may be identified.

Because self-reported height and weight might contain errors, using self-reported BMI results in some misclassification when allocating BMI categories, which can lead to biased risk calculations. Previous studies have indicated that the rate of misclassification might vary from 12% to around 20% [9].

Despite minor average differences, several people had clinically significant changes, suggesting that measurements should be taken in clinical practice and research with a clinical focus. Furthermore, these variations may cause incorrect inferences to be drawn about the outcomes of public health initiatives and policies. Specifically, a systematic underestimation of body mass index (BMI) in the elderly population may have consequences on determining the proportion of this group at risk of diseases including diabetes, hypertension, and functional impairment, as well as related costs to the healthcare system [10].

Self-reported measurements resulted in an underestimation of underweight categories and an overestimation of overweight categories, even though there was a strong correlation between the measured and personal evaluation of height, weight, and BMI in both boys and girls. Epidemiological studies may, however, utilise self-reported height and weight data to estimate participants’ fundamental anthropometric features; however, this is not feasible for clinical or therapeutic procedures [26].

Since it can be costly and problematic to acquire clinical measurements for every individual, especially in large-scale epidemiological studies, self-reported height and weight are frequently used for BMI estimation. Self-reports offer a quick, easy, affordable, and non-invasive way to obtain anthropometric data [11]. Despite the possibility that some people overestimate their height and underestimate their weight, prior research has demonstrated that self-reported height and weight may correspond favourably to measured measurements. In summary, this study demonstrates that although direct measurements are the best approach, self-reported weight and height information may be a reliable substitute, especially in extensive epidemiological investigations [5].

Researchers should be aware that people often overestimate their height and underestimate their weight, which results in an underestimation of BMI. This is especially true for women and people of Indian and Malay heritage. Therefore, it may be necessary to use caution when interpreting self-reported data to estimate the clinical state of overweight or obesity among individuals in Singapore [17]. Thus, self-reports’ potential and appropriateness should be assessed by clinicians and researchers in relation to their clinical or research needs.

### 4.4. Practical Considerations for Enhancing Reliability

Recent advancements in national health survey data gathering techniques have reduced costs and enhanced flexibility. When acquiring direct measurements is not feasible due to logistical, budgetary, or practical reasons, self-reported techniques may be employed. For instance, physical measures such as weight and height are expensive and time-consuming. Therefore, individuals may self-report their height and weight. Such self-reported metrics must be validated in order to ensure data quality and prevent bias resulting from incorrect categorisation [17].

The Bland–Altman approach suggests that the size of the differences between self-reported and measured weight and height values may have an impact on the agreement between them, albeit this effect appears to be minimal (β = −0.025). The variety of the discrepancies between the results under comparison was highlighted by the Bland–Altman test [15]. Although this method is not typically applied to measurement validation, it is important to assess the scope of any issues that may arise from using self-reported height and weight to estimate one’s weight status.

Key endpoints of the POEmaS trial, the validation study’s results, indicate that in trials that provide technology-enabled weight reduction therapies, the accuracy of self-reported data is equivalent to measured anthropometry [5]. The validity of the results provided through online platforms might be better understood by assessors of digital health treatments thanks to these findings. Additionally, it is helpful to let researchers know about digital health interventions when conventional anthropometry evaluation methods—like in-person or online questionnaires—are impractical or unavailable [5].

By comparing self-reports to measurements made by researchers directly, it has been discovered that self-reports are valid and dependable. It has been demonstrated that these self-reported anthropometric measurements hold up over time and are beneficial for epidemiological and health reasons for a minimum of 10 years [29]. In the field of healthcare, longitudinal research is both desperately needed and infamously hard to finance. Therefore, the data validity lifespan is very important, particularly when dealing with chronic illnesses like obesity. Therefore, using measurements that can be correctly performed without a researcher present will be crucial to dissemination and implementation studies. The researchers who create the programme and data analysis would not have the time or resources to visit every programme participant and collect trustworthy measures like anthropometrics while the programme is being distributed globally. Resolving this issue would require using precise self-reported data [29].

Since BMI calculation relies on self-reporting, it is critical to look at ways to collect data by using this method, which is more accurate. Redesigning survey questions to include more information on weight and height is one option that has been largely overlooked in the literature on public health. The literature on survey research has long recognised that the way questions are posed may significantly affect the answers that are received. Therefore, it would seem conceivable that phrasing the questions in a different way may increase the accuracy of self-reported height and weight.

Empirical data suggest that requesting precise information from participants—a process known as “priming”—improves the precision of delicate or invasive survey questions. Increasing the level of confidence regarding the confidentiality of the data is a promising way to improve the accuracy of self-reported weight and height [29]. This technique has been demonstrated to minimise misreporting. Despite the fact that most surveys offer these guarantees at the outset or throughout the informed consent procedure, further assurance before the height and weight questions are asked might enhance reporting.

Lastly, the framing effects could be significant. The process via which individuals view and conceptualise a problem is referred to as framing. Framing effects are when shifts in how an issue is presented lead to shifts in viewpoints [30]. Wording effects and context effects are two subcategories of framing effects. Context effects are the impact of the question’s asking context on survey replies. Wording impacts are related to the question’s wording. These effects have been noted for several different problems. Framing effects are typically examined in connection with opinions, but they may also be significant for other kinds of survey responses, such as self-reporting weight and height. However, not much research has looked at how they affect these kinds of concerns [29].

The findings of this systematic review emphasise the need for more study to assess plausible causes of variations in self-reported weight and height, as well as to create a practical and accessible formula to account for these biases in the community. Planning research designs and making inferences with self-reported anthropometric data in local epidemiology investigations will require this expertise.

Overall, our study provided valuable insights into the reliability and validity of self-reported weight and height data. However, some limitations were encountered during the research process. One notable limitation was the lack of comprehensive investigations into the sociodemographic, cultural, ethnic, and psychological factors influencing the variations between self-reported and measured weight and height. Another limitation was the lack of literature studies on the ways to minimise the influence of factors leading to differences in self-reported and measured data.

However, this systematic review holds significance in terms of providing a comprehensive overview of the factors influencing the reliability and validity of self-reported data. This can serve as a foundation for future research studies to explore alternative methods or ways for collecting anthropometric data to minimise biases associated with self-reporting and improve the accuracy of data collection. In order to improve the validity and reliability of self-reported anthropometric data, future research might concentrate on improving survey design and measuring methods. To enhance the accuracy of self-reported measures, survey questions that use priming techniques or framing effects may boost participants’ confidence and lessen social desirability bias.

## 5. Conclusions and Recommendations

Finally, the measurement of differences and validity problems between measured and self-reported weight and height presents evidence for a complex landscape which is created and affected by different factors. Although self-reporting is beneficial because it is convenient and cheap, the data may be biased due to low health awareness, the presence of social pressures, or the characteristics of each group people are from.

Though misclassification and biases may exist, self-reported measurements are still important indicators that complement the limited direct anthropometric data, especially in large-scale surveys with population groups that may be inaccessible. On the other hand, self-reported data should not be over-interpreted, particularly during clinical diagnosis, which could lead to the wrong classification of individuals’ health condition and the risks attached to it. Among practical considerations for ensuring the reliability, there are the evaluation of the population, the validation of the reported data, and the potential upgrading of the survey design and measuring technique to tackle the possible sources in bias.

Consequently, future studies should further explore the background of self-reported-data variability and seek ways to correct those biases, especially across different populations. This knowledge will help to refine study designs, increase data quality, and achieve correct conclusions in epidemiological studies, which are based on self-reported anthropometric data. As a result of all these challenges, self-reported procedures give us first-hand information on population health progress and should be used appropriately along with other direct measurements to obtain proper and accurate assessments.

## Figures and Tables

**Table 1:** Inclusion and exclusion criteria.

Item	Inclusion Criteria	Exclusion Criteria
BMI measurement	Studies that directly compare self-reported and objective measures	Studies that did not directly contrast self-reported and objective measures
Study design	Primary studies (observational or experimental study designs)	Secondary studies (reviews and meta-analyses)
	Case–control, prospective, retrospective, cross-sectional, qualitative, and RCT designs	Study designs not falling within the specified categories
BMI measurement tool	Utilisation of stadiometer, tape measure, portable or balance beam scale, or any other validated method for measuring weight or height	Studies not utilising the specified measurement methods or self-reporting methods
	Self-reporting methods in any format (e.g., interviewer-administered and self-complete)	Reports submitted by proxy, in which one person answers on behalf of another
Publication year	Studies published in the last 10 years	Studies older than 10 years
Geographical location	No limitations	Not applicable

**Table 2:** Summary and characteristics of the 10 included studies.

First Author, Year	Continent	Country	Study Design	Gender	Age	Sample Size	Self-Reporting Method	Difference between Self-Reported and Measured
Fry et al. 2022 [10]	Oceania	Australia	Quantitative: National Health Survey (2017–2018)	Male and female	65 years or over	3412	Interview	Compared with men, women underestimated weight more, by 0.75 kg. Age-related overestimations of height increased in general; individuals 85 years of age or older overestimated their height by 2.5 cm compared with those 65–69 years of age. The difference between the overestimates made by males and females was 0.9 cm.
Beleigoli et al. 2019 [5]	South America	Brazil	Randomised controlled trial	Male and female	18 and 60 years	1298 participants were randomised by a stratified randomised block design balanced by gender and category of body mass index	Questionnaire	The weight that was measured and that which was reported did not differ significantly (*p* = 0.13). The average difference between the measured and reported height was 0.4 cm (*p* < 0.001). This increased the likelihood that the measured weight was higher than the reported weight by one unit every BMI level.
Moreira et al. 2018 [15]	South America	Brazil	Quantitative: PNS Survey (2013)	A total of 52.9% of the individuals were women, and 77.1% were men	≥18 years	40,366	Questionnaire	ICC > 0.88 indicated a high degree of agreement between self-reported and measured body mass index, weight, and height.
Štefan et al. 2019 [16]	Europe	Croatia	Quantitative: cross-sectional	Boys (N = 134) and girls (N = 152)	Secondary-school students (11–16 years)	286	Self-administrated questionnaire	Boys and girls interpreted height and weight differently. These variations were insignificant, as demonstrated by Cohen’s D effect. For both boys and girls, the range of Pearson’s coefficient of correlation between self-reported and measured values was 0.95 to 0.97.
Ko et al. 2022 [11]	Asia	Korea	Raw data from the 2018 CHS	Male and female	19 years and older	214,640	Survey	The height that was self-reported was higher than the height that was measured, with men reporting 0.59 cm more and women reporting 0.71 cm more. In contrast, the self-reported weight was underreported compared with the measured weight; for both genders, this difference was 0.55 kg and 0.67 kg, respectively.
Chia et al. 2023 [2]	Asia	Malaysia	Quantitative: cross-sectional	Male and female	18 years and older	2781	Questionnaire survey	There were 0.4 kg and 0.4 cm discrepancies in the mean reported weight and height compared with the measured values.
Roystonn et al. 2021 [17]	Asia	Singapore (Chinese, Malay and Indian ethnicity)	Cross-sectional, epidemiological survey	Male and female	65 years and above	5132	Interview survey	Weight (0.8 kg) was underestimated, while height (0.2 cm) was overstated. The differences in weight (−0.95 kg) and height (0.35 cm) were greater in women.
Özdemir et al. 2020 [18]	Asia/Europe	Turkey	Quantitative: cross-sectional	304 males and 313 females	17–30 years	617 university students	Questionnaire	Males had a mean accuracy of 1.83 cm and females a mean accuracy of 2.42 cm for the overstated height. The underreported weight for males and females were 0.35 kg and −0.95 kg, respectively.
Hodge et al. 2020 [9]	North America	USA	Quantitative survey	712 men and 1817 women	30 to 65 years	2643	Self-administered enrolment survey	Men generally reported being 0.48 cm taller and 1.54 kg heavier than they actually were. Women reported being 0.16 centimetres taller and −0.88 kilogrammes lighter than they actually were.
Olfert et al. 2018 [6]	North America	USA	Quantitative: cross-sectional	Male and female	18–28 years	1562	Electronic survey	Of the individuals, 30.4% (*n* = 413) self-reported being within ±5.08 cm (2 inches) of their objectively measured height. Of the individuals, 75.1% (*n* = 996) self-reported being within ±2.3 kg (5 pounds) of their actual weight.

## Supplementary Materials

The following supporting information can be downloaded at: https://www.mdpi.com/article/10.3390/nu16111704/s1, Table S1: Databases and search strings, Figure S1: Traffic light plot for randomised controlled trial, Figure S2: Summary plot for randomised controlled trial, Figure S3: Traffic light plot for non-randomised observational studies, Figure S4: Summary plot for non-randomised observational studies.
