# Supplementary material for: Validity of Measured vs. Self-Reported Weight and Height and Practical Considerations for Enhancing Reliability in Clinical and Epidemiological Studies: A Systematic Review
Source: Nutrients. 2024 May 30;16(11):1704. doi: 10.3390/nu16111704 (PMC11175070; doi:10.3390/nu16111704)
Supplement: Supplementary file 1 [file nutrients-16-01704-s001.zip › nutrients-3000451-supplementary.pdf]

Table S1: Databases and Search Strings

Database	Search String
PubMed and Medline	("comparative validity" OR "validity comparison") AND (measured OR objective) AND (self-reported OR self-report) AND weight AND height AND (clinical OR medical) AND epidemiological AND ("practical strategies" OR interventions) AND reliability AND "anthropometric data collection"
CINAHL	TI ("comparative validity" OR "validity comparison") AND TI (measured OR objective) AND TI (self-reported OR self-report) AND TI weight AND TI height AND TI (clinical OR medical) AND TI epidemiological AND ("practical strategies" OR interventions) AND TI reliability AND TI "anthropometric data collection"

Risk of Bias Assessment

Supplementary Figures 1 and 2 depict the traffic light and summary plot for the randomized controlled trial by Du et al. (2019), completed using ROB-2.0. For non-randomized, observational studies, ROBINS-E tool was used to generate the traffic light and summary plots for the remaining 9 studies (Supplementary Figure 3 and 4).

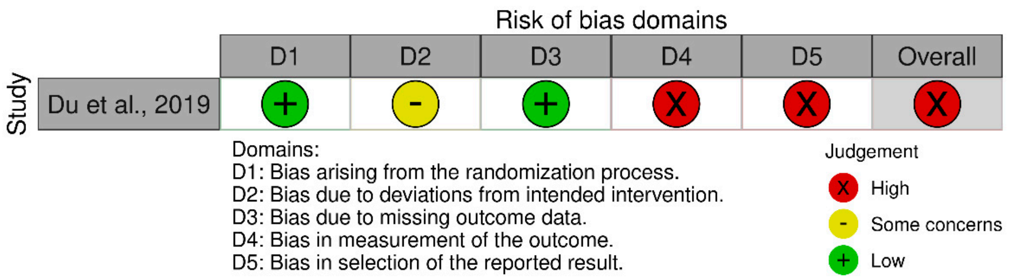

Figure S1: Traffic light plot for randomized controlled trial

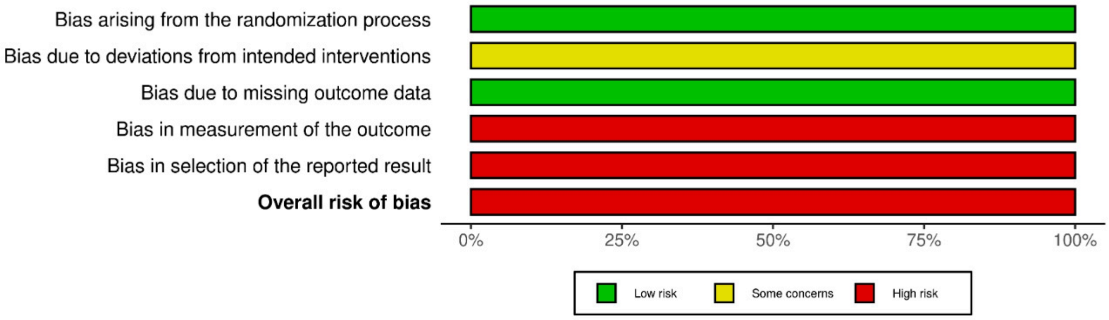

Figure S2: Summary plot for randomized controlled trial

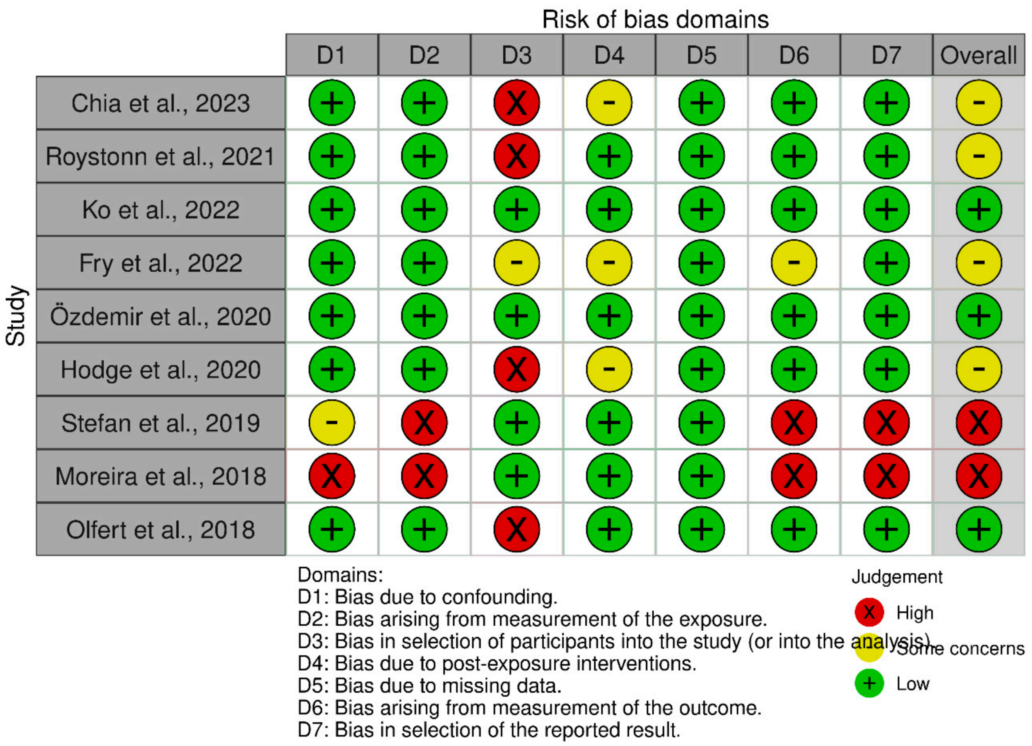

Figure S3: Traffic light plot for non- randomized observational studies

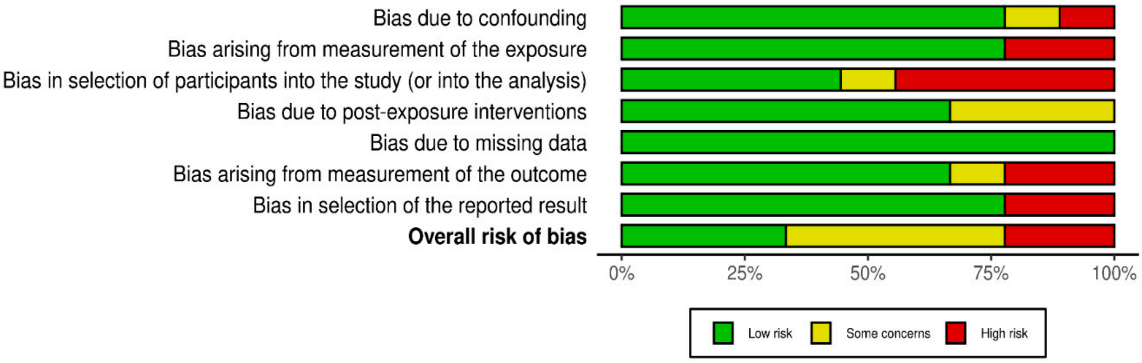

Figure S4: Summary Plot for non- randomized observational studies
